# Embedding Piezoresistive Pressure Sensors to Obtain Online Pressure Profiles Inside Fiber Composite Laminates

**DOI:** 10.3390/s150407499

**Published:** 2015-03-27

**Authors:** Maryam Kahali Moghaddam, Arne Breede, Christian Brauner, Walter Lang

**Affiliations:** 1 Institute for Microsensors, Actuators and Systems (IMSAS), University of Bremen, Otto-Hahn-Allee, NW1, 28359 Bremen, Germany; E-Mail: wlang@imsas.uni-bremen.de; 2 Faserinstitut Bremen e.V. (FIBRE), Gebäude IW3, Am Biologischen Garten 2, 28359 Bremen, Germany; E-Mails: breede@faserinstitut.de (A.B.); brauner@faserinstitut.de (C.B.)

**Keywords:** embedding, piezoresistive/capacitive pressure sensor, real-time pressure measurement, fiber composite laminate, infusion

## Abstract

The production of large and complex parts using fiber composite materials is costly due to the frequent formation of voids, porosity and waste products. By embedding different types of sensors and monitoring the process in real time, the amount of wastage can be significantly reduced. This work focuses on developing a knowledge-based method to improve and ensure complete impregnation of the fibers before initiation of the resin cure. Piezoresistive and capacitive pressure sensors were embedded in fiber composite laminates to measure the real-time the pressure values inside the laminate. A change of pressure indicates resin infusion. The sensors were placed in the laminate and the resin was infused by vacuum. The embedded piezoresistive pressure sensors were able to track the vacuum pressure in the fiber composite laminate setup, as well as the arrival of the resin at the sensor. The pressure increase due to closing the resin inlet was also measured. In contrast, the capacitive type of sensor was found to be inappropriate for measuring these quantities. The following study demonstrates real-time monitoring of pressure changes inside the fiber composite laminate, which validate the use of Darcy’s law in porous media to control the resin flow during infusion.

## 1. Introduction

The production of fiber composite materials is very expensive and time-consuming. In particular, the development process is often based on many trial-and-error experiments. This results not only in a long process development time, but also a waste of energy and material inputs. To circumvent this difficulty, experience-based approaches need to be replaced with knowledge-based methods. The aim is to realize stable processes, and to increase energy and material efficiency. In other words, there should be fewer required attempts and a lower quantity of wasted products. The critical step toward achieving a high-quality product is the successful impregnation of the fiber mat. By controlling the pressure and temperature of the process, the complete impregnation of the fibers will be achieved before initiation of the resin cure. This knowledge about the pressure and the temperature can be obtained by embedding sensors inside the fiber composite laminate, and thus acquiring real-time knowledge about the changes of these two parameters. The focus of this work is to measure the changes of the pressure in the laminate and to compare the measured data obtained from the sensor to the analytical calculation of the pressure.

Recently, various methods and sensors have been used to study the flow of resin in fibrous mat, including a DC-resistance sensor to determine the resin viscosity profile and using such a profile to maximize the flow of the resin and the impregnation of thick laminates [1]. Another method involves exploring the effect of fiber architecture on the quality of the final composite, which leads to discovery about the dependency of the permeability of the porous medium on the architecture of the fabrics. Small changes in the architecture of the preform or fiber misalignments result in unexpected resin flows and consequently void formation or dry spots [2]. Additionally, modeling of micro- and macroscopic flow using numerical calculations and working out the proposed flow equation in real time to determine the flow front shape of the resin has also been explored [3]. Modelling the fibers and tows microscopically to clarify the effect of the capillary force and viscous force on the formation of micro voids is another option [4]. Researchers have also investigated using a distributed fiber optic sensor array to observe the flow front position of the resin [5,6]. Another method involves verifying the flow front position by an ultrasonic torsional guided wave sensor for different fluids with different viscosities [7,8]. Finally, visually tracking the flow front of the resin in a transparent mold using cameras has been investigated [9].

As a result of the pressure gradient in the fluid, the resin flows through the laminate. Therefore, a pressure sensor provides more information about the physical changes of the pressure than just the arrival of the resin at the certain point, which can be detected by other sensors, including temperature or interdigital capacitive sensors [10,11]. Successful impregnation of the fibers can be assured by controlling the pressure and temperature before initiation of the resin cure, and accordingly, void formation will be reduced by pressure control in the production process [1]. Void growth will occur if the void pressure is higher than the real pressure of the resin, while it is in the liquid phase. The void pressure is naturally the volatile vapor pressure, while the resin pressure is hydrostatic pressure [12]. The purpose of this study is to provide a new method to measure pressure values inside the laminate in real time. By having knowledge about the pressure inside the laminate, especially for composite parts with thick or complex shapes, the proper pressure and temperature control will guarantee the complete impregnation of the fiber before the initiation of resin cure. This leads to high-quality products with a reduction in the amount of voids, microvoids and porosity. Additionally, resin flow simulations that are completely based on the pressure field can be validated by simply comparing the pressure values.

## 2. Experimental Procedure to Embed Pressure Sensor in Laminate

**Figure 1:**
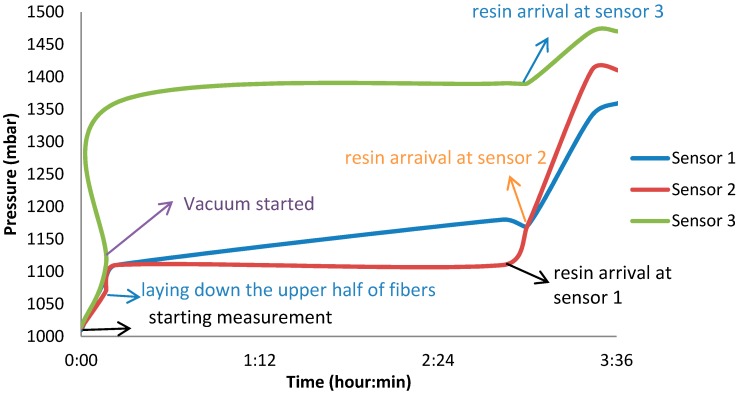
Real-time pressure profile inside the fiber composite laminate, measured by capacitive pressure sensors.

Three different experiments have been performed to track the pressure gradient in a fiber composite laminate, using capacitive and piezoresistive pressure sensors. The setups of the fiber laminates for all three experiments are similar, as described in Section 2.5. The first experiment was performed by embedding three capacitive pressure sensors in a glass fiber laminate (produced by Protron Microtechnique, www.protron-mikrotechnik.de). This type of the sensor measured a high parasitic capacitance when the upper layers of the glass fibers were laid on the sensors, when the vacuum pump started working, when the upper layers of the glass fibers compressed down, and also when the resin reached this sensing area (shown in Figure 1). In all cases, the sensors measured a pressure above the atmospheric pressure. After turning on the vacuum pump, the pressure inside the bag is required to be less than 1000 mbar. In fact, when the resin arrives to the sensor element, the pressure increases from the vacuum. The highest achievable pressure is atmospheric pressure (approximately 1000 mbar). This is due to the placement of the resin pot under room pressure, while the sensors measured a pressure higher than the atmospheric value upon the arrival of the resin.

**Figure 2:**
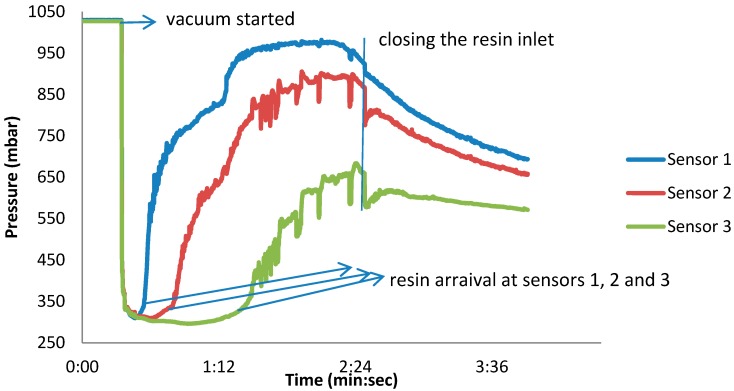
Real-time pressure profile inside the fiber composite laminate, measured by BOSCH pressure sensors.

The same experiment was repeated with a barometric pressure sensor (BMP 085, Robert Bosch GmbH, Germany). The sensors responded well to the initiation of vacuum (pressure drop from atmospheric pressure to vacuum) and to the arrival of the resin (pressure increase) as it is shown in Figure 2. For various reasons, this type of sensor also could not be embedded in the composite for measuring the pressure changes during the infusion. Some of these reasons included:

- Based on the data sheet of the Bosch BMP 085 pressure sensor, the operating range is 300–1100 mbar, while the vacuum infusion is typically performed at lower pressure.
- The sensor and the ASIC are integrated on a rigid PCB board, which significantly disturbs the uniformity of the final product.
- The full accuracy of the sensor is 0–65 °C (operating range 0–85 °C), which is low for autoclaving or Resin Transfer Molding (RTM) processes.

Finally, the third test has been performed by using a piezoresistive pressure sensor (PPS) (the term piezoresistive pressure sensor (PPS) refers to the sensor die, which is the bare pressure sensor element), which can withstand up to 150 °C for long periods (at least 180 °C for 24 h was verified by the authors). To reduce the mechanical distortion of the composite by the embedded sensor, the sensors have been wire bonded to a thin, flexible and high temperature stable board.

### 2.1. Wire Bonding PPS on Flexible Board

To obtain the pressure from a PPS embedded in laminate, the sensor needs to be glued and wire bonded to a board. The measured pressure will be transferred to evaluation boards (external to the laminate) *via* the soldered wire to the other side of the board. In this particular experiment, we fabricated a thin, flexible board. After calibration of the sensors for different pressures (0–1020 mbar), they were embedded into the laminate. These different steps are explained in the following sections.

### 2.2. Fabrication of Flexible Board

**Figure 3:**
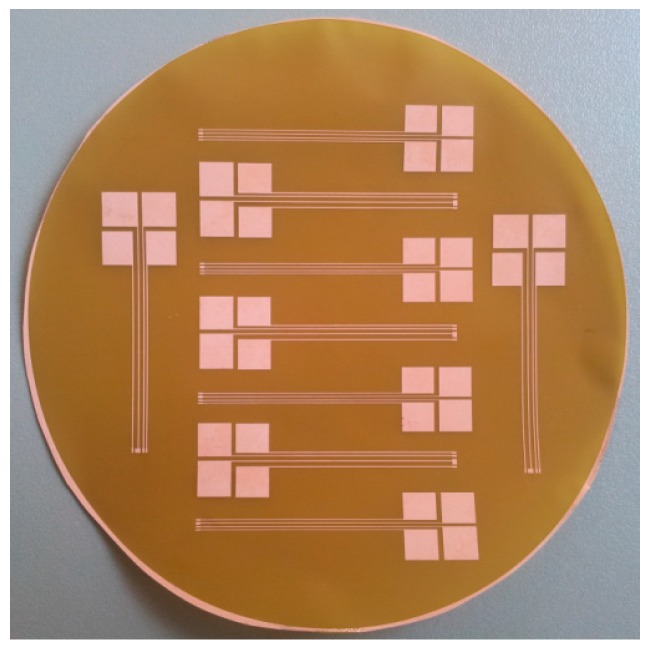
The Pyralux wafer after structuring the copper coating.

For the fabrication of the flexible board, to which the piezoresistive pressure sensor was glued and subsequently wire bonded, a polyimide foil with a thickness of 25.4 µm covered on both sides with 35 μm copper (Pyralux- a DuPont^TM^ product) was used. The foil was cut in a circular shape with a diameter of approximately 10 cm; we refer to this as the Pyralux wafer. Then, it was covered by Ti-Prime to improve the adhesion between the photoresist and copper.The Pyralux wafer was then spin coated with 1.8 µm positive photoresist. The curing of the Ti-Prime and positive photoresist was performed on a hot plate for 2 min at temperatures of 120 °C and 100 °C, respectively. The next step involved exposing the Pyralux-wafer to UV light and developing the photoresist. The copper was then etched in a solution of Alketch for approximately 13 min. Figure 3 shows a photograph of the Pyralux-wafer after structuring the copper.

**Figure 4:**
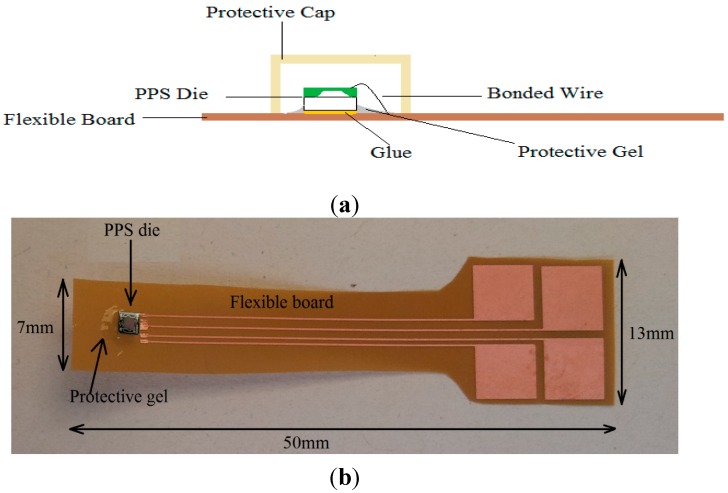
(**a**) Schematic and (**b**) a photograph of the glued piezoresistive pressure sensor (PPS) die on a flexible board.

Next, the Pyralux wafer was cut into a single flexible board and the piezoresistive pressure sensor was glued on that board. This was followed by wire bonding the PPS die to the pads on the flexible board. Figure 4 depicts the schematic and a photograph of the flexible board with mounted and wire bonded sensor. Finally, the bonded wires were covered with HIPEC^®^ Q1-4939 on the flexible board side to prevent corrosion or breakage of the bonded wires during the embedding process. The gel was cured in a convection oven at 150 °C for 120 min.

### 2.3. Preparing the Sensor’s Cap

To prevent the stimulation or even damage to the sensor membrane by compression of the fibers under vacuum, a mold was prepared to produce a suitable cap for the sensor. Glass fibers and the resin RTM6 were used. A mold and vacuum bag were set up in an oven with a temperature of 180 °C for 2 h to cure the resin. The side view of the sensor covered with such a cap is shown in Figure 4a. Two parallel sides of the cap were left open to let the resin flow onto the sensor membrane.

### 2.4. Calibration of the PPS on the Flexible Board

**Figure 5:**
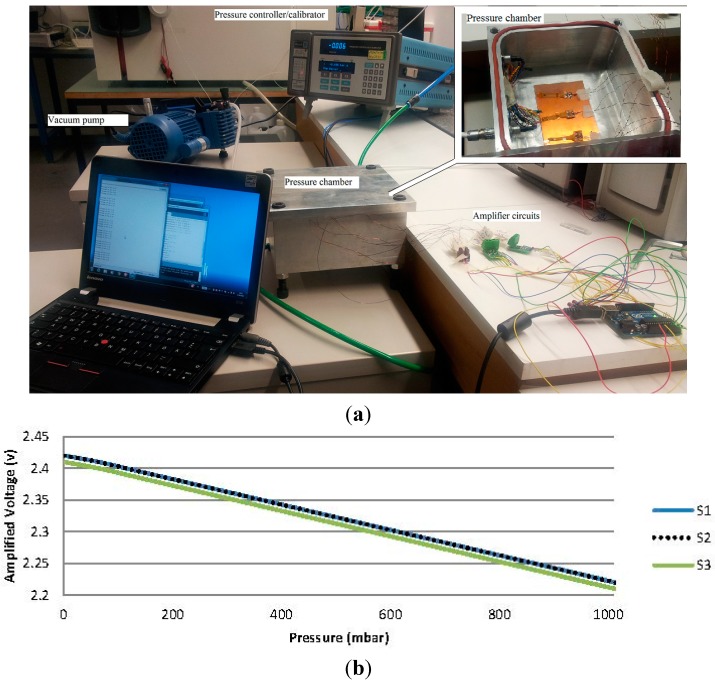
(**a**) Calibration setup consists of pressure chamber, vacuum pump, amplifier circuits, evaluation boards and a laptop; (**b**) calibration graph of piezoresistive pressure sensor for the range of 0–1020 mbar.

In the next step, the wires were soldered to the pads on the flexible board (right side pads, Figure 4b), connected to the amplifier and evaluation board, and all of the flexible boards were laid into the pressure chamber to calibrate them at various pressures ranging from 0–1020 mbar (Figure 5a).

Calibration graphs for the sensors are shown in Figure 5b. All of the sensors showed completely linear and similar values upon increasing and decreasing the pressure in the range of 0–1020 mbar with a step size of 50 mbar. An amplifier circuit was used to enhance the output voltage of the sensors by a ratio of 30.

In this work, the PPS die produced by First Sensor Technology GmbH (Standardline STARe) was used. The dies were wire bonded to be connected on a flexible PCB (Figure 4). This flexible PCB was thin (25.4 µm) with high temperature stability (up to 220 °C). The sensor and the flexible board remained inside the laminate, and the evaluation board for data measurement was on the outside of the laminate.

### 2.5. Embedding the PPS Die on Flexible Board in Fiber Composite Laminate

Embedding the PPS in a laminate is initiated by placing half of the glass fiber plies (bidirectional glass fiber is used here) on a metallic plate and fixing the sealing tape around them. Next, the PPSs with their flexible boards are laid down on the glass fibers, and the second half of the plies are placed on top of the first half and the sensors. Subsequently, the resin inlet and outlet pipes, suction pipes (connected to vacuum pump), and the vacuum bags are set. Next, the resin (two component epoxy resin of the EPIKOTE™ Resin MGS^®^ RIMR 035c type is used in this study) can be infused, and after complete impregnation of the fibers, the metallic plate is heated up to initiate the resin cure.

**Figure 6:**
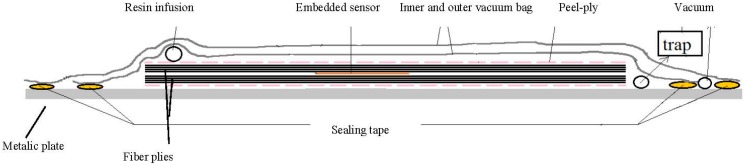
A typical vacuum bag setup.

A simplified schematic of a vacuum bag setup is shown in Figure 6. The resin is infused into the inner bag through the resin infusion pipe and suctioned out of it by a vacuum pump on the other side. The outer bag is also connected to the vacuum pump to secure the vacuum.

**Figure 7:**
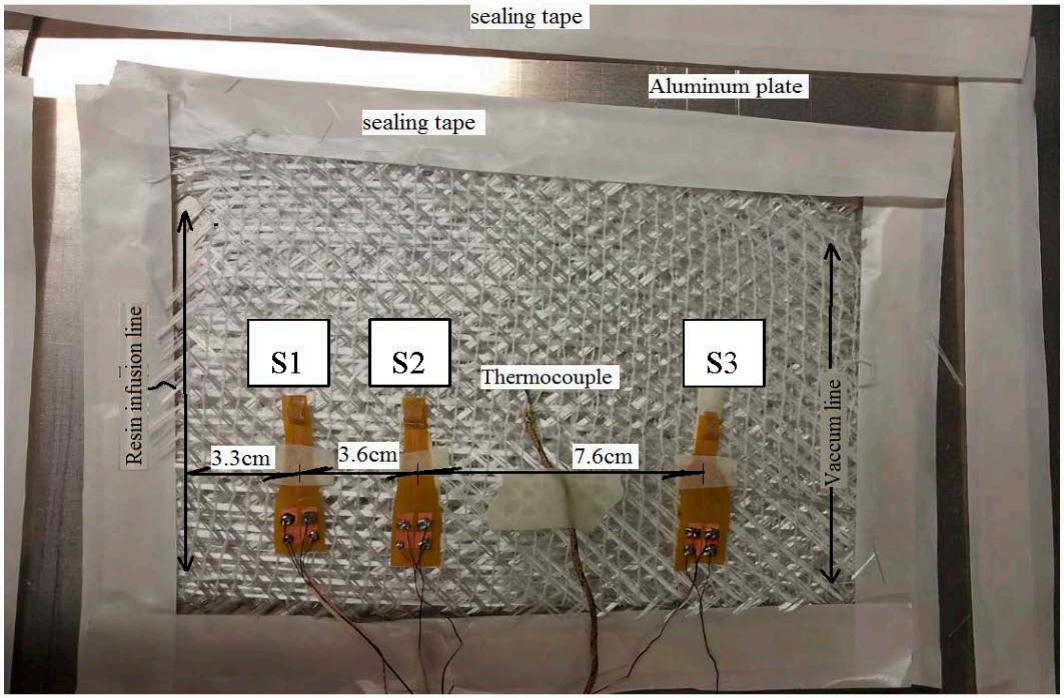
Embedding of three PPSs mounted on flexible board in the glass fiber laminate.

Three piezoresistive pressure sensors mounted on top of flexible boards were placed on three plies of glass fibers (Figure 7). The distance of the first sensor from the resin infusion line was 3.3 cm, while the distance between Sensors 1 and 2 was 3.6 cm. Sensor 3 and the vacuum line were at a distance of 4 cm. The distance between Sensor 2 and 3 was 7.6 cm. A thermocouple between Sensor 2 and 3 measured the temperature inside the laminate. The setup was mounted on an aluminum plate. Subsequently, to initiate the resin cure, the aluminum plate was heated from the bottom. Since the entire thickness of the fiber composite laminate was very thin (less than 2 mm in comparison with the real production’s thickness, which could be around 4 cm), the composite can be considered as a 1D surface.

### 2.6. Analytical Calculation for the Pressure Gradient during Infusion

#### Theory and Methodology of the Resin Pressure Distribution during Fiber Impregnation in a Composite Laminate

In modeling the resin flow inside the fibrous mat, the mat is considered as a porous medium, and Darcy’s law applies:

(1) $$\vec{\nu }=-\frac{K}{\eta }\;\nabla \overset{\to }{p}$$

where $\vec{\nu }$ is the volume average velocity, K is the permeability tensor, η is the resin viscosity, and *p* is the fluid (here resin) pressure [13]. For incompressible fluids, the continuity equation states:

(2) ∇.ν=0

The combination of Darcy’s law and the continuity equation results in:

(3) $$\nabla .(\frac{K}{\eta }\;\nabla \overset{\to }{p})=0$$

**Figure 8:**
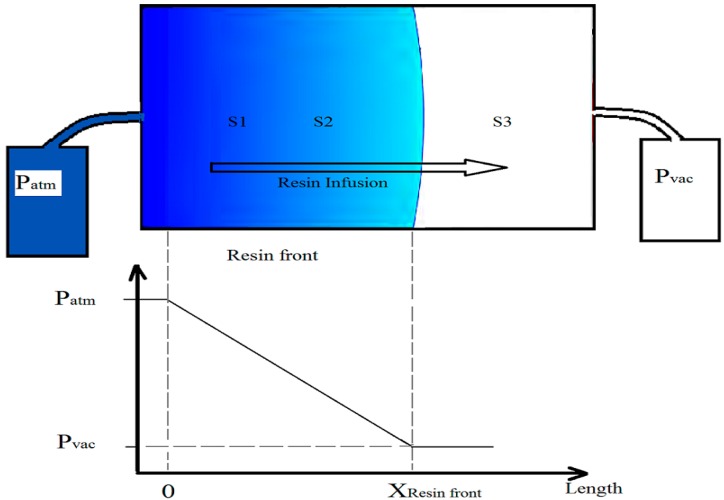
Pressure gradient between the infusion line and the vacuum line in the laminate.

Equation (3) is the source of fluid pressure field determination in fiber composite laminates. During the infusion, the fiber mat is partially filled by air and partially by the resin (Figure 8). Since the resin viscosity is significantly higher than the air viscosity, the loss of pressure from the resin front to the outlet (which is not yet filled with the resin) can be omitted. Thus, vent pressure can be considered as a boundary condition at the flow front position. By defining the material properties K and η, Equation (3) will give us the unique solution for the pressure distribution [13].

The changes of pressure from the infusion source of the resin (infusion line in Figure 7) to the resin front (X_Resin Front_ in Figure 8) drops linearly. At the infusion side, the pressure is equal to the atmospheric pressure, which is the pressure of the resin pot at room pressure (approximately 1013 mbar). At the resin front, the pressure is equal to the vacuum pressure, since the vacuum pump is connected to this side.

### 2.7. Comparison Between the Measured and Analytical Calculated Pressure during Infusion

The fiber mat setup in this experiment had dimensions of 18 × 12 cm (Figure 7), where the inlet and outlet of the resin were placed along the short opposite sides of the mat. Since the thickness of the mat was very small compared with the length and the width of the mat, the mat can be considered a 1D surface. The infusion time is defined as the period of time from opening the the resin inlet (to fill the vacuum bag) until the entire fiber mat is wetted by the resin. The infusion time was measured to be approximately 9 min. We used MATLAB to calculate Equation (3). We performed some iterations based on the recorded time and place of the resin front, which values are listed in Table 1.

**Table 1:** Time, resin front and PPS sensor’s position.

Time (min)	X (cm)	D (cm)
1	3	(S1) 3.3
2	6	(S2) 6.9
3	9.5	
4	12	
5	13.5	(S3) 14.5
6	15	
7	16.5	
8	17.5	
9	18.5	

Note: X (cm) is the front of the resin, and it is the length over which the resin swept through the fiber mat, and D (cm) is the distance between the sensors and the infusion line.

**Figure 9:**
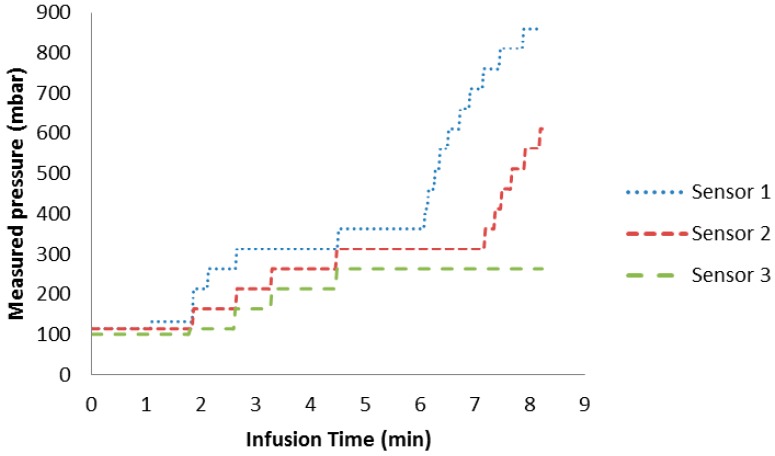
Measured pressures by the embedded PPSs in glass fiber laminate during infusion time.

**Figure 10:**
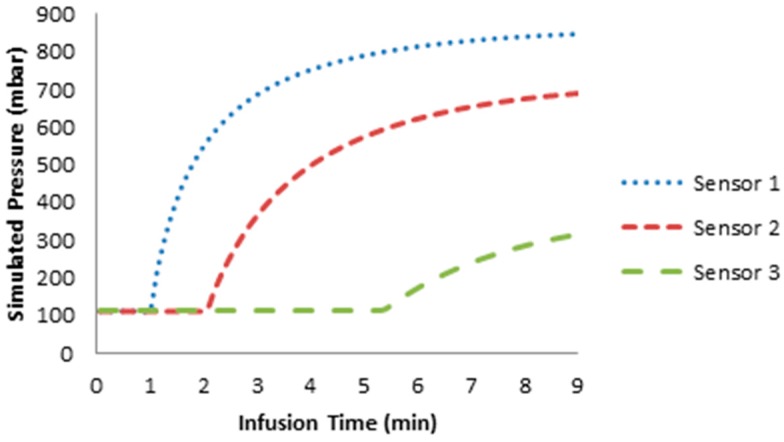
Simulation of the pressure based on Darcy’s law.

Figure 9 shows the pressure measured with the embedded sensor during the infusion time. Figure 10 depicts the analytical calculated pressure based on Darcy’s law. The embedded PPSs detect the arrival of the resin. There is an agreement between the model and the measured pressure at the end of the infusion time. This shows that the flow behavior can be accurately measured by the integrated pressure sensor array. Concerning the build-up curves of the pressure at each sensor, we find a characteristic difference, which will be the subject of future theoretical and experimental investigation.

## 3. Results and Discussion

**Figure 11:**
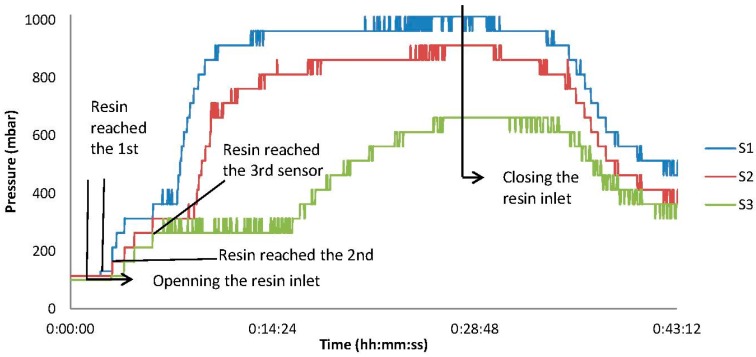
Real-time pressure profiles inside the fiber composite laminate at infusion time.

The embedded piezoresistive pressure sensor accurately tracked the initiation of vacuum and the pressure development throughout the infusion. The resin pot outside the vacuum bag was under the room pressure conditions (approximately 1013 mbar). When the infusion started, there was a pressure gradient from the infusion line to the vacuum line, as shown in Figure 8. The pressure in the infusion line is the atmospheric pressure and in the vacuum line, it is vacuum pressure, which depends on the pump capability and the entrance of air to the bags. The air, and subsequently, the extra amount of the resin, were suctioned out *via* the vacuum line. The infusion is based on the pressure difference between the inner bag (vacuum pressure) and outer side of the bag (atmospheric pressure). The measured pressure, at Sensors 1 and 3, show this pressure difference at the inlet and outlet of the resin inside the inner bag. While Sensor 2 (that has been placed in between Sensors 1 and 3 and closer to Sensor 1) shows a trend between Sensors 1 and 3. In the case of pressure ranges, this trend was mostly similar to Sensor 1 (Figure 11). In the other words, as shown in Figure 8, the changes of pressure over length is linear. This means the sensors near the infusion line will measure a higher pressure than sensors near the vacuum line during the infusion.

Figure 11 depicts the pressure changes from the initiation of vacuum, to infusion of the resin, and to closure of the resin inlet (the resin inlet is closed when the pressure does not change for approximately 5 min for all three sensors). Initially, the vacuum pump decreased the pressure in the bag down to 110 mbar; the vacuum pressure is stabilized in this range. The resin infusion valve was opened. The progress of the resin flow from Sensor 1 to Sensor 3 can be seen as an increase of the pressure measured by the sensors. This means that the embedded pressure sensors detect the infusion of the resin. To summarize, a more filled volume means a smaller pressure gradient. This directly leads to a smaller resin flow velocity. This fact is of primary concern, especially for large or thick laminates in which the reduction of the resin front speed may result in dry sections or unsaturated spots. This will significantly affect the quality of the products. To avoid this, the resin flow needs to be modeled, and this model needs to be validated by real-time measurements obtained by the embedded sensors. By achieving such a validated model of the resin flow, the trial-and-error-based development of fiber composite laminate production will be replaced by a knowledge-based method.

The resin front infiltrates Sensors 1 to 3 after opening the resin valve with a 1, 2 and 6 min delay, respectively. By considering the distances between sensors and the infusion line (shown in Figure 7), the velocity of the resin flow from Sensors 1 to 2 was 3 cm/min, and from Sensors 2 to 3 it was 1.9 cm/min. These are calculated from the division of the swept length over the elapsed time. In this test, the speed of the resin was reduced by a factor of 0.63 (1.9/3) from the interval of Sensors 1 and 2 to the interval of Sensors 2 and 3.

## 4. Conclusions/Outlook

In this study, capacitive and piezoresistive pressure sensors were embedded in a glass fiber composite laminate to observe the pressure changes in the laminate during resin infusion. The results showed that the fiber impregnation can be tracked by measuring the pressure inside the fiber laminate composite using embedded piezoresistive pressure sensors, while the capacitive pressure sensors measured the parasitic capacitance from the dielectric properties of the glass fibers and resin. Pressure tracking is very important when the mold has a complex shape or the laminate is thick, and consequently, the flow behavior of the resin is complicated. Knowledge-based methods have to be utilized to determine the flow comportment of the resin. To develop such a knowledge-based method, sensors must be used. As a result, by varying pressure and temperature in the laminate, the curing can be initiated after ensuring the complete impregnation of the fibers. In particular, for a part with a complex shape, the critical point must be identified, and pressure sensors should be placed there. In this way, before heating the resin to initiate curing, the arrival of the resin and impregnation of the fibers is ensured by measuring the pressure at those critical points.

By having real-time information about the pressure inside the laminate, and monitoring the pressure changes, the number of voids (formed in the spaces between the tows), microvoids (formed in the spaces between the fibers), dry spots, and resin-rich areas will be reduced. This will result in improved quality of the final fiber composite products and reduced wastage products. This allows the production costs and consumed energy to be reduced significantly.

By obtaining the pressure field from inside the laminate, resin flow simulation can be validated. Future work will involve embedding interdigital planar capacitive sensors to track the curing of the resin. Moreover, some mechanical tests (interlaminar shear strength and fatigue tests) could be performed on a composite specimen with and without embedded sensors to determine the distortion caused by the embedded sensor in the composite.

The outlook suggests the direction of replacing the wire bonding method with flip chip technology. This will eliminate the wire debonding that occurs during measurement and the need for a protective cap. Moreover, it will result in smaller sizes of the embedded sensing equipment inside the composite laminate, which entails the usage of less mechanically invasive instruments. The next step could involve embedding the pressure sensor on a flexible board in different composite production processes, such as high-temperature or high-pressure ones (autoclave processes and presses such as prepreg and thermoplastic materials).
